# A neuro-inspired computational framework for AGI: predictive coding, active inference and free energy minimisation

**DOI:** 10.3389/fnhum.2026.1917153

**Published:** 2026-10-08

**Authors:** Lioba C. S. Berndt, Alexander D. Shaw

**Affiliations:** 1 Computational Psychiatry and Neuropharmacology Lab, Department of Psychology, Faculty of Health and Life Sciences, University of Exeter, Exeter, United Kingdom; 2 School of Physiology, Pharmacology and Neuroscience, University of Bristol, Bristol, United Kingdom

**Keywords:** active inference, AGI, free energy principle, GI, predictive coding

## Abstract

This paper offers a conceptual synthesis of foundational principles from theoretical neuroscience [i.e., predictive coding, the Free Energy Principle (FEP) and variational inference] as a biologically grounded framework for artificial general intelligence (AGI). We adopt this predictive-coding and FEP perspective as our organising framework. It is one of several current accounts of neural computation and not all available evidence is consistent with it. These approaches characterise the brain as a hierarchical inference system that continuously updates beliefs and selects actions to minimise uncertainty and surprise. In contrast to conventional AI systems, biological agents engage in active, generative inference within dynamic, uncertain environments. We argue that this inference-based architecture and not behavioural outputs alone, plausibly underlies the adaptability, generalisation and resilience of natural intelligence. We outline a neuro-inspired computational framework built on hierarchical generative models, scalable variational inference (i.e., Variational Laplace) and Active Inference. Finally, we contrast this approach with dominant deep learning paradigms and discuss its hypothetical implications for building interpretable, adaptive and autonomous machine intelligence.

## 1 Introduction

The human brain remains the only known system capable of general intelligence (GI), defined here as the ability to flexibly interpret, model and act within dynamic, uncertain and novel environments through a unified mechanism by which perception, action and learning are continuously co-optimised. While formal definitions vary, GI is typically characterised by context-sensitive reasoning, adaptive learning and goal-directed action across multiple domains (Legg and Hutter, 2007). A growing body of work in theoretical neuroscience suggests that a key contributor to this flexibility is not memorised patterns or fixed rules, but the brain's capacity for inference. Specifically, theories such as predictive coding (Rao and Ballard, 1999; Friston, 2005) and the Free Energy Principle (FEP) (Friston, 2010; Bogacz, 2017) propose that perception, action and learning arise from the hierarchical minimisation of prediction errors via approximate Bayesian inference. In this view, the brain is a generative model that continuously predicts sensory inputs, updates internal beliefs in response to discrepancies and selects actions that minimise expected future surprise. Importantly, FEP and predictive coding remain influential theoretical accounts among several competing views of neural computation and the evidence on its specific biological implementation is mixed (see discussion in Section 2 on this).

This predictive loop supports both epistemic behaviour (reducing uncertainty through exploration) and instrumental behaviour (pursuing preferred outcomes), achieved by internally simulating possible futures and evaluating them according to expected free energy (Parr et al., 2022). The underlying dynamics are managed by a variational principle: the brain minimises variational free energy (a tractable bound on surprise) which unifies learning, adaptation and action selection under uncertainty.

Meanwhile, artificial intelligence (AI) has made rapid progress in areas such as transformer-based large language models (LLMs), diffusion-based generative models and reinforcement learning agents (Vaswani et al., 2017; Ho et al., 2020; Brown et al., 2020).

Despite these breakthroughs, current AI systems achieve generalisation primarily through statistical pattern matching and what appears to be structured reasoning can emerge from memorised or interpolated patterns in training data (Mirzadeh et al., 2025; Zhao et al., 2025). Performance deteriorates under even moderate distribution shifts, including in multimodal settings (Neuhaus et al., 2026). While there is evidence that LLMs develop implicit representations of environmental structure (Kulveit et al., 2023), these fall short of the explicit, action-conditioned generative models that would support robust uncertainty quantification and online adaptation (Qazi et al., 2025).

In this paper, we synthesise the computational principles underlying brain function (predictive coding, dynamical systems modelling and active inference) as a candidate, biologically grounded framework for constructing general-purpose intelligent agents. We argue that such agents should not just reproduce intelligent behaviour, but embody the same self-organising, inference-based principles hypothesised to underlie human cognition.

We first review how hierarchical predictive coding and active inference support perception, action and learning in cortical dynamics. We then formalise the same objective through the FEP, relate variational free energy to the evidence lower bound and present a minimal Active Inference pong agent. Building on that account, we outline a neuro-inspired AGI architecture, contrast this inferential approach with dominant deep learning paradigms and characterise the gap between amortised offline training and continual hierarchical inference. We close with implications for neuro-inspired machine intelligence.

## 2 The brain as a model of general intelligence

Biological intelligence arises not from memorised rules or static mappings, but from the brain's ability to infer latent causes, predict sensory consequences and adapt behaviour accordingly. Predictive coding offers a plausible computational account of how this is achieved (Rao and Ballard, 1999; Friston, 2005). It posits that the brain maintains hierarchical generative models that continuously predict incoming sensory input and minimise the discrepancy between predicted and observed signals. This framework views perception, cognition and action as outcomes of approximate Bayesian inference embedded in a dynamical system (Friston, 2009). Under this assumption, the brain does not only react to the world but models it, anticipates it and selects actions that reduce uncertainty about it.

### 2.1 Hierarchical generative models and approximate bayesian inference

The brain is organised into a hierarchical architecture spanning both anatomical and functional levels in which each level generates predictions about the activity of the level below. Sensory signals are explained away by these top-down predictions and only residual errors (i.e., the unexpected components of sensory input) are propagated upward. This message-passing architecture supports approximate Bayesian inference over latent variables **z**, given sensory observations **x**, under a joint generative model (Friston, 2010):


$$\begin{array}{l} p\left(\text{x},\text{z}\right)=p\left(\text{x}|\text{z}\right)p\left(\text{z}\right) \end{array}$$ (1)

Because exact Bayesian inference is intractable in realistic settings, the brain is assumed to represent a variational posterior *q*(**z**) that approximates the true posterior *p*(**z**|**x**). Inference then proceeds by minimising a variational free energy (Friston and Stephan, 2007):


$$\begin{array}{l} F\left[q\right]=\text{KL}\left[q\left(\text{z}\right)||p\left(\text{z}\right)\right]-{𝔼}_{q\left(\text{z}\right)}\left[\log p\left(\text{x}|\text{z}\right)\right] \end{array}$$ (2)

Minimising free energy ensures that internal beliefs are both accurate (i.e., maximise the likelihood of observed input) and parsimonious (i.e., remain close to prior expectations). This provides a principled trade-off between accuracy and complexity, which is essential for adaptive intelligence (Friston, 2010; Bogacz, 2017).

Neurobiologically, predictive coding is thought to be implemented within canonical cortical microcircuits, which exhibit a consistent layered (laminar) and column-like (columnar) structure across sensory, associative and motor areas of the cortex (Bastos et al., 2012; Douglas and Martin, 2004). This architecture supports the bidirectional flow of information central to predictive coding. In these circuits, pyramidal neurons in the superficial layers (layers 2 and 3) are classically proposed to encode prediction errors (signals reflecting discrepancies between expected and actual sensory input), though this specific laminar and regional localisation is contested, as discussed below. These neurons are proposed to send forward projections to higher cortical areas, primarily targeting layer 4 of downstream regions. By contrast, deep-layer pyramidal neurons (layers 5 and 6) are thought to encode top-down predictions, and are proposed to project back to lower hierarchical levels, targeting both superficial layers and layer 4 interneurons, thereby modulating the gain of ascending prediction errors. Interlaminar inhibitory interneurons such as somatostatin-positive (SST) and parvalbumin-positive (PV) cells have been proposed to further refine this process by regulating the precision, or weighting, of prediction errors through modulatory control of excitatory neurons.

We note that this account represents one particular theoretical perspective on cortical computation and that the precise biological implementation of predictive coding remains debated. Recent analyses of neuronal spiking activity suggest that genuine prediction-error signals may emerge in prefrontal rather than early sensory cortex, challenging the classical predictive-coding account of sensory hierarchies (Gabhart et al., 2025). Direct neural implementations of the free-energy principle itself have been demonstrated more concretely elsewhere: canonical neural networks with Hebbian plasticity have been shown to perform active inference (Isomura et al., 2022) and free-energy minimisation has been empirically validated *in vitro* using cultured cortical neurons (Isomura et al., 2023).

On this account, this laminar organisation supports recursive message passing across hierarchical levels: prediction errors ascend to update beliefs, while predictions descend to explain away sensory input. On this view, the anatomical segregation of error and state representations across layers would enable dynamic inference over multiple timescales and levels of abstraction – from basic sensation to higher-order cognition. Crucially, this architecture is proposed to provide a neurobiological substrate for attention, precision modulation and context-sensitive gain control within predictive coding frameworks (Friston K., 2008; Shipp, 2016).

### 2.2 Predictive coding for perception

In the predictive coding account of perception, the brain is cast as a generative system that infers the hidden causes of its sensory inputs (Rao and Ballard, 1999; Friston, 2005). Each level of the cortical hierarchy encodes beliefs about latent variables in the level below, generating top-down predictions that are compared to incoming signals. When predictions fail to fully explain the input, the residuals (prediction errors Equation 3) are propagated upward (Friston, 2009). These errors update the internal model, refining beliefs about the underlying causes. This iterative, bidirectional message passing implements approximate Bayesian inference in real time (Bogacz, 2017).

Let *s* denote sensory observations and *z* the latent causes at a given hierarchy level. This is the same factorisation as in Equation 1, specialised to scalar notation. Because exact inference of *p*(*z*|*s*) is generally intractable, the brain maintains a variational approximation *q*(*z*), which is updated to drive prediction error toward zero. The prediction error ε can be written as:


$$\begin{array}{l} \varepsilon =s-ŝ\left(z\right) \end{array}$$ (3)

where ŝ(*z*) is the predicted sensory input based on current estimates of *z*. Assuming that both the variational posterior *q*(*z*) and the likelihood relating *z* to *s* are Gaussian, free energy minimisation leads to a gradient descent update of the latent causes (Friston K., 2008):


$$\begin{array}{l} \frac{dz}{dt}\propto \frac{\partial \varepsilon }{\partial z}\cdot {\text{Σ}}^{-1}\cdot \varepsilon \end{array}$$ (4)

Here, Σ denotes the precision (inverse variance) of the prediction error (Equation 4). Precision determines the influence of prediction errors on belief updating, effectively weighting the reliability of incoming sensory data (Parr et al., 2022).

**Example: predictive coding in visual perception**

Consider a simple visual scene in which a person views a cup on a table. Higher-level visual areas (e.g., inferotemporal cortex) generate abstract predictions such as “object with a handle” or “familiar graspable shape” (Muckli et al., 2015). These predictions are sent down the hierarchy, shaping expectations for the patterns of activity in lower-level areas (e.g., orientation edges in V1). If the incoming sensory input matches these expectations, prediction error remains low. However, if the object is partially occluded or appears unusual (e.g., a broken cup or an unexpected angle) prediction errors arise in early visual areas and are sent upward. These errors are crucial: they signal a discrepancy and prompt higher-level areas to update their beliefs until the model settles on the most likely explanation (e.g., “damaged cup”).

In this way, perception operates not as passive registration, but as the brain's best guess about the hidden causes of ambiguous, noisy input, allowing for rapid adaptation to new or unexpected situations.

Consistent with this account, recent computational modelling suggests V1 may predict, rather than simply discount, the illuminant in colour constancy, supporting a predictive role for this stage of the visual hierarchy (Gao and Li, 2026).

### 2.3 Action as inference: active inference

Notably, predictive coding is not restricted to perception. Within the framework of Active Inference, action selection is cast as an inferential process driven by the same imperative that governs perception: the minimisation of expected free energy (Friston et al., 2010). In this view, agents do not passively infer the causes of their observations but actively select policies that make future observations more predictable and less surprising.

The expected free energy *G*(π) (Equation 5) under a policy π can be decomposed into epistemic and instrumental components, capturing both uncertainty reduction (exploration) and goal-directed behaviour (exploitation). One common formulation expresses this as:


$$\begin{array}{l} G\left(\pi \right)={𝔼}_{q\left(\text{x},\text{z}|\pi \right)}\left[\log q\left(\text{z}|\pi \right)-\log p\left(\text{z}|\text{x},\pi \right)\right] \end{array}$$ (5)

Thus, it encourages the agent to seek actions that simultaneously reduce uncertainty about hidden states and achieve preferred outcomes (Parr and Friston, 2019; Da Costa et al., 2023). Perception and action are effectively unified under a single inferential scheme: the brain continually infers not only what is happening, but what it should do to bring about desirable and predictable states of affairs.

Unlike traditional reinforcement learning, which relies on externally defined reward signals and policy optimisation, Active Inference derives behaviour from an internal generative model and an imperative to minimise variational free energy. In this framework, goals and preferences are encoded as prior beliefs about preferred states and behaviour emerges from the drive to realise these states while reducing uncertainty. This makes Active Inference naturally suited to continual, adaptive control in uncertain environments, without requiring an externally defined scalar reward signal, though preferences, priors and model structure must still be specified by hand. Modern reinforcement-learning frameworks can themselves incorporate learned environment models (M. Moerland et al., 2023) and uncertainty-aware, information-seeking exploration objectives (Ladosz et al., 2022).

**Decomposing expected free energy**

Under Active Inference, agents select actions that minimise the expected free energy *G*(π), which captures both uncertainty reduction (epistemic value) and goal fulfilment (instrumental value). A common decomposition is:

$$G\left(\pi \right)=\underset{\text{Epistemic value (uncertainty reduction)}}{\underset{︸}{{𝔼}_{q\left(\text{x}|\pi \right)}\left[D_{\text{KL}}(q\left(\text{z}|\text{x},\pi \right)∥q\left(\text{z}|\pi \right))\right]}}+\underset{\text{Instrumental value (preference satisfaction)}}{\underset{︸}{{𝔼}_{q\left(\text{x}|\pi \right)}\left[-\log p\left(\text{x}\right)\right]}}$$

The first term encourages actions expected to reduce uncertainty about hidden states, thereby supporting epistemic exploration, whereas the second term encourages outcomes that are consistent with prior preferences and therefore supports instrumental goal pursuit.

Together, these components drive both exploration and exploitation without requiring an explicit reward signal.

### 2.4 Cognition as dynamical inference in state space

Rather than making isolated decisions or computations, the brain operates as a dynamical system: its activity is constantly changing and evolving over time (Rabinovich et al., 2006). At any moment, the pattern of neural activity can be represented as a point in a high-dimensional space, where each dimension corresponds to the activity of a different neuron or neural population. As the brain processes information, this point traces out a path, or trajectory, through the space. These trajectories reflect how the brain continuously updates its beliefs about the hidden causes of sensory input. This ongoing process is driven by prediction errors and is influenced by the brain's level of uncertainty about its current beliefs (Friston K., 2008; Friston, 2010).

Such recurrent neural dynamics, whether oscillatory, chaotic or metastable, can be formalised as solutions to differential equations that model the temporal evolution of beliefs (Equation 6). In continuous time, this is captured by:


$$\begin{array}{l} \frac{d\mu }{dt}=f\left(\mu ,\text{Σ},\text{x}\right)-\nabla_{\mu }F \end{array}$$ (6)

Here μ and Σ denote the mean and covariance of the agent's Gaussian belief, q(z)=N(z;μ,Σ) (formalised fully in Section 3). The first term *f*(μ, Σ, **x**) describes how beliefs would evolve over time based on prior expectations or natural dynamics. Essentially, how the brain expects things to change without new sensory input. The second term acts as a corrective force, adjusting beliefs to reduce prediction errors and improve the fit between the brain's model and actual sensory input (Parr et al., 2019).

These dynamics have been hypothesised to be implemented by specific biological substrates, though the mapping remains interpretive. Cortical microcircuits are thought to implement the message passing required for hierarchical inference. Thalamocortical loops are proposed to regulate the gain and precision of these messages through rhythmic synchronisation, and neuromodulatory systems (such as noradrenaline and dopamine) are similarly considered to modulate uncertainty by adjusting the precision weighting of prediction errors (Fiorillo et al., 2003; Bastos et al., 2012). On this account, cognition emerges as a continuous, embodied and context-sensitive process of inference. Here, embodiment means closed-loop, action-conditioned coupling between agent and environment, in which actions change future sensory input (not a specific biological or physical form). This closed-loop coupling is necessary for AGI as characterised here, since actions that do not alter future observations give the epistemic component of active inference nothing to act on. In this view, the brain functions as a dynamical generative system, continuously inferring both its own internal state and the state of the world. This precision-weighted balance between top-down and bottom-up inference has recently been shown to account for autonomous shifts between focused attention and mind-wandering in hierarchical variational recurrent networks (Oyama et al., 2025). A related account models V1 orientation adaptation as an update to the internal “state” of a spatial receptive-field model (Gao and Liu, 2022).

## 3 The free energy principle and variational laplace as inference engines

Section 2 motivated variational free energy as the objective for hierarchical inference in the cortex. The FEP uses the same variational formalism but shifts the explanatory target to any adaptive system (biological or artificial) that persists by coupling to an environment through sensory exchanges. Such systems are taken to maintain generative models and minimise variational free energy so as to remain consistent with observations and prior expectations, thereby bounding surprise and supporting viability in fluctuating conditions (Friston, 2010; Friston and Stephan, 2007; Bogacz, 2017). Thus, the arguments below apply equally to cortical hierarchies and to engineered agents. Sensory inputs **x** and latent states **z** enter through the joint model and variational objective in Equations 1, 2, in that form, minimising F[q] trades off accuracy against complexity (fitting observations while keeping the approximate posterior close to the prior).

### 3.1 Free energy as a unified objective and the ELBO

Importantly, the variational free energy corresponds to the negative evidence lower bound (ELBO) used in machine learning, so both disciplines optimise the same tractable surrogate for model evidence (Parr et al., 2022; Blei et al., 2017). Rearranging terms gives:


$$\begin{array}{l} \log p\left(\text{x}\right)\geq {𝔼}_{q\left(\text{z}\right)}\left[\log p\left(\text{x}|\text{z}\right)\right]-\text{KL}\left[q\left(\text{z}\right)||p\left(\text{z}\right)\right]=-F\left[q\right] \end{array}$$ (7)

Here **x**, **z**, *q*(**z**) and *p*(**z**) follow the definitions in Equations 1, 2, log*p*(**x**) is the log model evidence. Equation 2 rearranges directly into Equation 7 by expanding the KL term in F[q] and applying Bayes' rule to *p*(**z**|**x**) [see Blei et al. (2017) for the full derivation.]

Thus, minimising variational free energy is equivalent to maximising the ELBO. While machine learning typically uses the term ELBO, the FEP often speaks of variational free energy to emphasise thermodynamic and biological readings of the same bound (Parr et al., 2022).

### 3.2 Variational laplace: a scalable engine for dynamical inference

While the Free Energy Principle defines the objective an agent still requires a concrete algorithm to achieve this in practice. Standard optimisation methods like gradient descent can be slow or biologically implausible. A more efficient and neurally inspired alternative is Variational Laplace, an inference scheme that forms the basis of neuroimaging tools such as Dynamic Causal Modelling (DCM) (Friston et al., 2007; Friston K. J., 2008).

Variational Laplace operates under a key simplifying assumption that the approximate posterior distribution over hidden states is Gaussian (Equation 8). In this formulation, an agent's beliefs are represented by a mean vector μ and a precision matrix Σ^−1^, encoding both what is believed and how certain those beliefs are:


$$\begin{array}{l} q\left(\text{z}\right)=N\left(\text{z};\mu ,\text{Σ}\right) \end{array}$$ (8)

The method efficiently updates these beliefs by approximating the local curvature of the free energy landscape using a second-order Taylor expansion. This allows it to compute both the direction and step size for updates (akin to the Newton–Raphson method) enabling faster and more stable convergence than simple gradient descent.

In continuous time, this results in a gradient flow over beliefs,


$$\begin{array}{l} \frac{d\mu }{dt}=-\nabla_{\mu }F\left(\mu \right), \end{array}$$ (9)

consistent with the dynamical view of inference in Section 2 (cf. belief trajectories driven by prediction error). The agent refines μ by moving along the free-energy landscape rather than performing a one-shot optimisation (Equation 9).

Variational Laplace offers a scalable and interpretable inference engine that is argued to support this kind of online adaptation, making it a candidate approach for modelling biological intelligence as well as building adaptive artificial agents. As we show in later sections, this approach can be applied directly to active perception and control tasks, including our toy Active Inference agent for Pong.

### 3.3 Learning and adaptation under the free energy principle

Learning, i.e. updates to generative parameters θ, operates on a slower timescale than the state and policy inference described above, but follows the same objective (Friston et al., 2009) meaning fast dynamics infer hidden causes and select actions, while slower dynamics adjust θ to reduce free energy accumulated over time.

### 3.4 From principle to practice: active inference in 2D Pong

We implemented a minimal Active Inference agent to control a paddle in a 2D Pong-style environment (Figure 1). In this environment, a ball moves within a bounded square arena at constant speed, bouncing elastically off the walls. Its hidden state includes its position and velocity in both dimensions. A paddle, height 0.2 and fixed at the right-hand wall, moves up, down, or stays still each step in increments of 0.05 and does not affect the ball's physics. The agent observes the full state with added Gaussian noise (SD 0.02).

**Figure 1:**
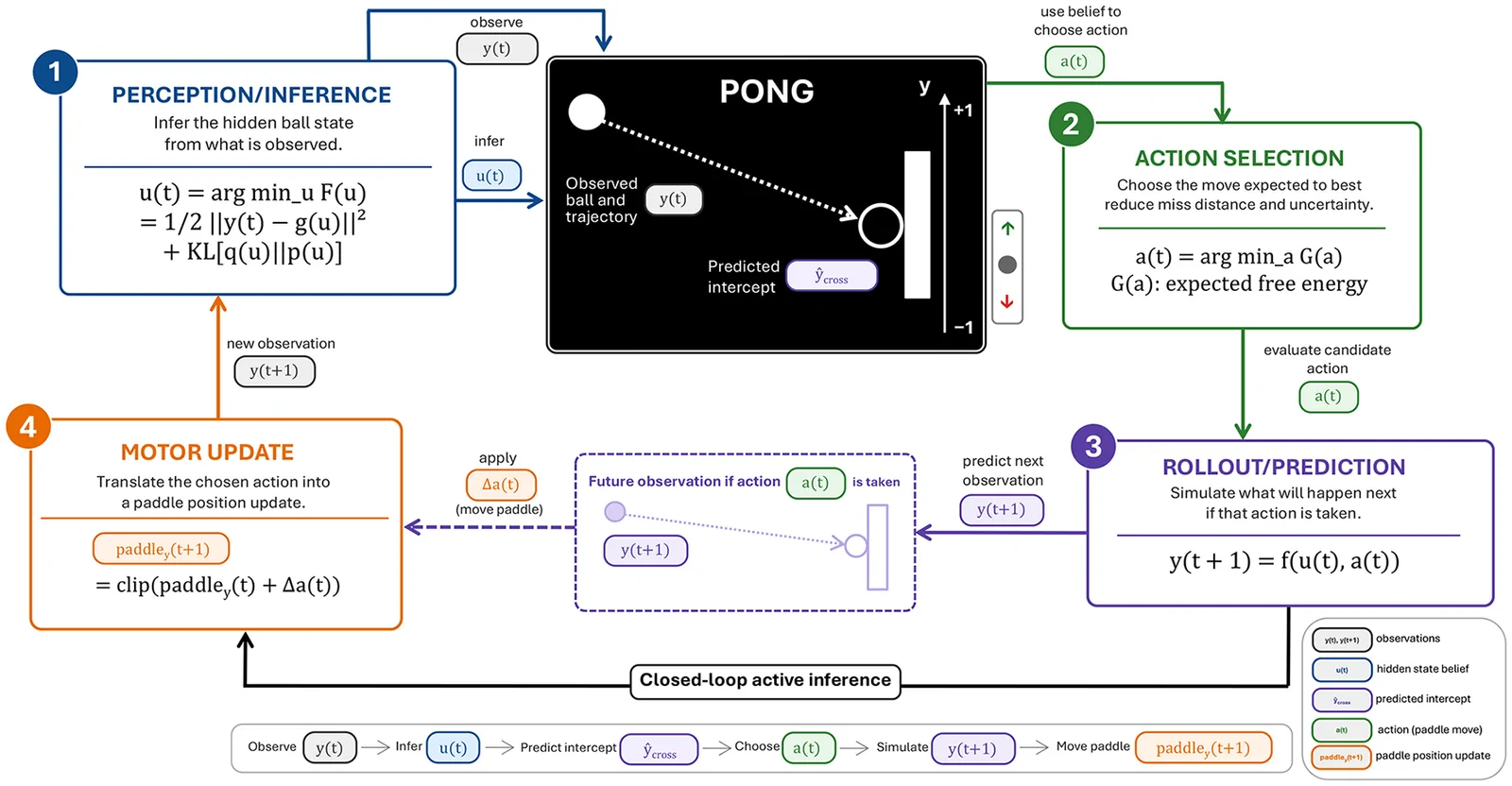
Closed-loop active inference control in a Pong environment. A variational Laplace scheme infers a belief, μ(*t*), about the underlying ball dynamics by minimising variational free energy. This objective combines sensory prediction error, ||*y*(*t*)−*g*(*u*)||^2^, with a prior regularisation term, KL[*q*(*u*)||*p*(*u*)]. The inferred state is used to estimate the likely intercept point of the ball with the paddle axis, ŷ_cross_. Action selection evaluates candidate paddle movements and selects the action *a*(*t*) that minimises a short-horizon pragmatic cost, *G*(*a*), favouring moves that reduce expected miss distance. The ball state is rolled forward under the fixed dynamics, û(*t*+1) = *f*(μ(*t*)), while the candidate action updates only the paddle position, paddle_*y*_(*t*+1) = clip(paddle_*y*_(*t*)+Δ*a*(*t*)). Coloured variable tags indicate how key quantities are passed between stages: observations *y*(*t*), inferred beliefs μ(*t*), predicted intercepts ŷ_cross_, actions *a*(*t*), predicted future ball states û(*t*+1) and updated paddle positions. Here a one-step action is treated as a degenerate policy, π = (*a*). Because the observation model is full-state and action-independent, the epistemic term is constant across candidate actions, so *G*(*a*) denotes the pragmatic cost used for action selection.

The agent infers the ball's hidden state from its observations at each time step using Variational Laplace, under a hand-specified generative model with an identity observation function, so the observation is treated simply as a noisy copy of the true state (Friston et al., 2007; Bogacz, 2017). Explicitly, for hidden ball state the observation mapping is *g*(*u*_*t*_) = *u*_*t*_. All structural quantities are hand-specified. During online inference, the approximate posterior belief over the ball state is updated while the model parameters and assumed dynamics remain fixed.

Given this belief, the agent evaluates a short set of candidate paddle moves by rolling the inferred ball state forward under the fixed ball dynamics while separately rolling the paddle position forward under each candidate action. The action does not affect the ball's dynamics. It only affects the future paddle position and candidate actions are scored by the expected miss distance between the predicted ball intercept and the predicted paddle position. This is the pragmatic, preference-driven component of expected free energy. The epistemic component is absent here since the agent already observes the full state at low noise, thus, seeking further information would not change which action is chosen. A natural extension would let this choice also account for the agent's residual uncertainty instead of its best estimate alone and would only require an epistemic term if observations depended on the action taken, for instance, through occlusion or a partial view. Unlike reinforcement learning approaches, which typically learn policies from external reward signals via trial-and-error, this agent uses a model-based, inference-driven strategy. Its behaviour emerges from the minimisation of variational free energy and this pragmatic cost, enabling online adaptation and uncertainty-aware decision making without an externally defined reward signal, though the preference weighting used above was itself hand-specified.

This closed loop, inference informing action and action changing the agent's future sensorimotor relation to the environment, is a concrete instance of the general cycle described in Section 2, where belief updating follows the same free energy minimisation given in Equation 2.

To evaluate this behaviour, we ran the agent over 50 episodes. Its position inference matched the observation noise [root mean square error (RMSE) 0.0200 ± 0.0003] and it tracked the ball closely (mean tracking error 0.0399 ± 0.0013), intercepting it on 236 of 250 approaches (94.4%). A random-action policy given identical observations tracked far worse (0.3432 ± 0.0259) and intercepted only 48 of 250 times (19.2%).

These results reflect belief updating within a correctly-specified model, not robustness to distribution shift. To test this, we ran the agent under three *post-hoc* violations of its assumed dynamics, introduced at *t* = 500 within otherwise identical 50-episode runs a threefold speed increase, inelastic bounces (restitution *e* = 0.6) and added downward acceleration (0.002/step) with the generative model and rollout assumptions held fixed throughout. The pre-shift half of each episode is identical across conditions, giving a matched baseline, so post-shift differences reflect the physics change alone.

The agent's overall behaviour was sensitive to these shifts, but this sensitivity appeared to reflect degradation in the control rollout rather than in state inference. Position RMSE stayed at the noise floor (0.0199 ± 0.0005) throughout, since inference only ever fits the current observation. Paddle control, however, degraded in proportion to how far the fixed rollout's assumptions departed from the true dynamics. The tracking error roughly tripled and hit rate fell from 0.90 to 0.50 under gravity, where the rollout is wrong at every step, vs. a milder drop to 0.85 under the speed jump, where updated observations partly compensate for the stale model.

The framework is therefore not robust to distribution shift in general. In practice, this means an accurate state estimate does not guarantee good behaviour if the rollout model used to plan ahead is wrong. Applications where dynamics may change would need either to monitor the rollout's predictions against incoming observations, flagging misspecification or to replace the hand-specified rollout with a learned one that can update as evidence accumulates (Section 4).

The full implementation is available at https://github.com/alexandershaw4/VFE_2D_Pong.

### 3.5 General intelligence in biological agents

On the account developed here, GI in biological agents does not come from learning specific input-output mappings or storing task-specific solutions alone. Instead, it is proposed to emerge from a capacity for continual, structured inference, dynamically estimating latent causes, updating beliefs over time and selecting actions to minimise future uncertainty. From this perspective, the brain's solution to GI is not a fixed algorithm, but a dynamical system for approximate inference in uncertain and nonstationary environments. Hierarchical generative models, precision-weighted prediction errors and temporally extended action selection together form a biologically grounded architecture for flexible cognition (Clark, 2013). Crucially, we propose that predictive coding, Active Inference and FEP offer more than a descriptive theory of neural computation. We suggest they may offer a candidate mechanistic template for constructing artificial systems that can reason, plan and adapt in a manner analogous to the situated, embodied processes of human agents, though this remains hypothetical. If GI requires the ability to model hidden causes, update beliefs online and act to shape future outcomes, then these frameworks may serve as a candidate framework for AGI. Consistent with this view, recent theoretical work proposes that recursive precision-control across a hierarchical system underlies forms of epistemic agency and flexibility reminiscent of general intelligence (Laukkonen et al., 2025).

## 4 Toward a neuro-inspired AGI architecture

In this section we translate the preceding framework into design principles and an architecture sketch for neuro-inspired AGI.

### 4.1 Key design principles

Building on this coupled account of perception, action and learning (Section 3.5), we outline concrete design principles. A hierarchical organisation allows high-level states to send predictions downward while lower levels return precision-weighted prediction errors upward (Friston, 2005). Beliefs μ(*t*) are updated online through variational inference (for example, Variational Laplace) (Friston et al., 2007; Bogacz, 2017) and precision estimates are adjusted dynamically so that uncertain signals are weighted appropriately (Parr and Friston, 2019). Actions are selected by minimising expected free energy *G*(π), balancing epistemic information gain with preference-consistent outcomes. Parameters θ are then updated over longer timescales to reduce cumulative free energy. Structure learning, revising the generative model's form itself, remains an active area of ongoing work (Neacsu et al., 2026; Tazawa and Isomura, 2025), and stable, general-purpose mechanisms for realistic settings remain unresolved. Hierarchical generative models (Section 2) provide a basis for compositional abstraction, since higher levels encode reusable, more abstract causes shared across contexts and for transfer, insofar as these abstract representations can be re-used when lower-level statistics change. Precision-weighted inference and expected free energy minimisation are proposed to offer a mechanism for flexible goal formation, since preferences are encoded as prior beliefs that could in principle be re-specified without retraining the generative model itself. This, however, has not been demonstrated in the implementation presented here. Online parameter updating (Section 3) offers a possible route to continual skill acquisition, although whether it reduces forgetting in large-scale sequential learning remains to be empirically demonstrated. Long-term memory and hierarchical planning over extended time periods are not directly demonstrated by the architecture or the Pong example presented here and remain open problems. Existing Variational Laplace implementations operate over comparatively short inference windows and small state spaces and scaling hierarchical generative models to support planning over long horizons, while retaining tractable inference, is an unresolved computational challenge for the framework as a whole.

### 4.2 Architecture sketch

The pseudocode below condenses Sections 2, 3 into one operational cycle: infer hidden states, evaluate policies via expected free energy, act and update parameters (Millidge, 2020). Hierarchical prediction-error passing is included when multiple representational levels are used.

**Pseudocode: active inference loop for neuro-inspired AGI**

**Initialize:**

θ← generative model parameters

μ← initial belief over hidden states

Σ← initial precision (inverse covariance)

**Loop over time** ***t* = 1, 2, …*****n*****:**

1. **Observe sensory input:**
  *y*_*t*_← sensory observation from environment
2. **Infer hidden states via Variational Laplace:**

$\mu_{t}\approx \arg\min_{\mu }F\left(\mu \right)$

$F\left(\mu \right)=\frac{1}{2}||y_{t}-g\left(\mu \right){||}^{2}+\text{KL}\left[q\left(\mu \right)||p\left(\mu \right)\right]$

3. **Update precision:**
  Σ_*t*_← function of current residual and belief uncertainty
4. **Evaluate actions via expected free energy:**
  *G*(π) = *𝔼*_*q*_[log*q*(**z**|π) − log*p*(**z**|**x**, π)]
5. **Select and execute action:**

$a_{t}=\arg\min_{a}𝔼\left[G\left(\pi \right)\right]$

  Execute *a*_*t*_ in environment
6. **Update generative model parameters:**

$\theta \leftarrow \theta -\eta \nabla_{\theta }F\left(\theta \right)$

7. **Propagate hierarchical updates (if applicable):**
  Update higher and lower levels via prediction errors

*Note:* This pseudocode is intended as a conceptual sketch. Several steps (i.e., evaluating the expected free energy over all possible future trajectories, or performing stable online updates to the generative model's parameters) have significant computational and theoretical challenges. In practice, these operations would require substantial approximations, heuristics, or architectural constraints to become tractable. Nevertheless, this schematic illustrates the integrated nature of perception, inference, learning and action in Active Inference frameworks, grounded in the minimisation of variational free energy.

## 5 Deep learning, predictive coding and inferential gaps

While the principles of hierarchical inference provide a compelling candidate framework for general intelligence, the trajectory of modern AI has followed a significantly different path. Contemporary systems are dominated by deep learning paradigms, particularly convolutional neural networks (CNNs) in vision (LeCun et al., 2015) and Transformer-based architectures in natural language processing (Vaswani et al., 2017; Brown et al., 2020). These systems achieve remarkable performance by learning statistical regularities from massive datasets, yet they primarily implement high-dimensional, static mappings. This computational style becomes clear when we inspect the core update rules used in each architecture.

In CNNs, a canonical layer update takes the form


$$\begin{array}{l} h^{\left(l\right)}=\sigma \left(W^{\left(l\right)}*h^{\left(l-1\right)}+b^{\left(l\right)}\right), \end{array}$$ (10)

where *h*^(*l*)^ is the activation of layer *l*, *W*^(*l*)^ and *b*^(*l*)^ are its learned weights and bias, * denotes convolution, and σ(·) is a fixed nonlinearity. Each layer applies a learned transformation to the previous layer's representation. Transformers follow the same general logic, with self-attention typically expressed as


$$\begin{array}{l} \text{Attention}\left(Q,K,V\right)=\text{softmax}\left(\frac{QK^{⊤}}{\sqrt{d_{k}}}\right)V, \end{array}$$ (11)

where *Q*, *K* and *V* are the query, key and value matrices computed from the input via learned projections, and *d*_*k*_ is the key dimension used to scale the dot product.

Equations 10, 11 illustrate that, in both architectures, the parameters [including the mapping *W*^(*l*)^, *b*^(*l*)^ and the projections underlying *Q*, *K*, *V*] are fixed once training ends, so that within a forward pass no explicit posterior over latent causes is inferred or updated. We do not intend this to characterise all contemporary deep learning as lacking any form of adaptation. Recurrent architectures, autoregressive Transformers, world models and in-context or test-time adaptation mechanisms do carry state or update behaviour across a sequence or task. Indeed, it has been argued that Transformer attention implicitly commits to a non-Markovian generative model in which context is used to predict future observations (Parr et al., 2025). However, even in these cases the parameters of the mapping itself, i.e. *W*^(*l*)^, *b*^(*l*)^ and the attention projections, remain fixed at inference time, so that adaptation occurs within a learned function and not through explicit inference over a generative model, as described in Sections 2, 3. This reliance on fixed mapping parameters creates a fundamental inferential gap between artificial and biological intelligence as characterised here. Diffusion models offer a further point of contrast. Widely used for image generation (Ho et al., 2020), they learn, via a variational bound, to reverse a fixed noising process by predicting and removing noise across many steps. Once trained, however, the parameters of this denoising function are fixed, and sampling proceeds by repeatedly applying this fixed function to a noise sample, rather than through online inference over an explicit posterior belief updated from new observations, as described in Sections 2, 3.

Although modern models exhibit capability fragments (including in-context adaptation and emergent world modelling) these often remain decoupled capacities. Relative to the precision-weighted prediction errors emphasised in cortical inference, uncertainty estimates in deep models can degrade substantially in novel environments (Ovadia et al., 2019).

Predictive coding and Active Inference (Friston, 2010; Bogacz, 2017) are hypothesised to mitigate these limitations, through explicit inference over latent states, precision-weighting of prediction errors and a unified objective linking perception, learning and action. This suggests several potential, though as yet unproven, advantages. Precision-weighting is argued to enable context-sensitive behaviour by balancing sensory evidence against prior expectations (Feldman and Friston, 2010), and the unified perception-action loop is argued to allow behaviour to emerge from the same inferential machinery used for state estimation (Friston et al., 2010). However, FEP-inspired architectures are not inherently immune to the limitations they are meant to address: variational recurrent networks and related models can themselves exhibit sensitivity to distribution shift, miscalibrated uncertainty and forgetting, and our own Pong results (Section 3) show degraded control under exactly this kind of shift. Whether the mechanisms above yield a robust advantage over conventional deep learning at scale therefore remains an open empirical question rather than an established finding. Predictive coding models typically rely on vertical, hierarchical organisation, whereas Transformers have been argued to achieve comparable, hierarchical-like processing through lateral, contextual integration instead (Queißer et al., 2025).

Biologically inspired frameworks also face substantive limitations. Like deep learning, they often rely on predefined state spaces and incur substantial computational overhead when scaled to complex, real-world generative models. While they offer a strong theoretical framework for situated agency, robustness to distribution shift is not itself established by this framework, as our own Pong results illustrate, and biologically inspired approaches have not yet matched deep learning's raw performance on mainstream, large-scale benchmarks. At present, their empirical strengths are most pronounced in neurobiological modelling (Friston et al., 2007; Friston K. J., 2008; Parr et al., 2019) and constrained control simulations (Millidge, 2020; Tschantz et al., 2020). For AGI research, the central challenge may therefore be integration rather than replacement: combining deep learning's scaling power with coherent, uncertainty-aware inferential architectures that better support GI.

## 6 Conclusion

Theoretical neuroscience provides more than a metaphorical lens; it offers a principled computational perspective on general intelligence. By casting perception, action and learning as processes of variational inference, predictive coding and the FEP suggest a unifying account of information processing across cognitive domains (Friston, 2010).

Unlike many AI approaches that rely primarily on static architectures trained on fixed datasets, neuro-inspired systems grounded in Active Inference aim to infer causes, forecast consequences and adaptively select actions to minimise uncertainty and pursue goals (Millidge et al., 2021). In that sense, they emphasise generative, predictive and epistemically guided behaviour rather than purely reactive mappings.

It is important to note that these are aims motivated by the theoretical framework. Our own worked example shows that state inference can remain accurate even as downstream control degrades once the environment departs from the agent's assumed dynamics, underscoring that the advantages proposed here are hypotheses for future work and not established results.

The vision presented here is one of convergence: narrowing the gap between modelling the mind and building machine intelligence with the potential for greater flexibility, adaptiveness and context sensitivity in uncertain environments, by integrating insights from cortical computation, hierarchical generative models and variational optimisation, contingent on resolving the scaling and robustness challenges discussed above.

Ultimately, progress toward artificial general intelligence may depend less on mimicking behavioural outputs alone and more on developing architectures that capture at least some of the inferential machinery underlying biological intelligence.

## Data Availability

The original contributions presented in the study are included in the article/supplementary material, further inquiries can be directed to the corresponding author.
